# Chronic periodontitis and risk of lung cancer: a nationwide cohort study

**DOI:** 10.3389/fonc.2024.1413590

**Published:** 2024-06-28

**Authors:** Bo-Guen Kim, Hyun Lee, Sun-Kyung Lee, Sun Young Paik, Seo-Hyoung Yun, Chang-Joo Park, Yoomi Yeo, Tai Sun Park, Ji-Yong Moon, Tae-Hyung Kim, Jang Won Sohn, Sang-Heon Kim, Ho Joo Yoon, Dong Won Park

**Affiliations:** ^1^ Department of Internal Medicine, Hanyang University College of Medicine, Seoul, Republic of Korea; ^2^ Department of Mathematics, College of Natural Sciences, Hanyang University, Seoul, Republic of Korea; ^3^ Division of Oral & Maxillofacial Surgery, Department of Dentistry, Hanyang University College of Medicine, Seoul, Republic of Korea

**Keywords:** chronic periodontitis, lung cancer, epidemiology, risk, periodontitis

## Abstract

**Background:**

The impact of long-term chronic periodontal conditions on the risk of lung cancer could not be accurately evaluated. Our aim was to provide more evidence on the connection between chronic periodontitis (CP) and lung cancer using a nationwide dataset.

**Methods:**

This study used data from the Korean National Health Insurance Service National Sample Cohort. We enrolled 72,658 individuals with CP (CP cohort) between 2005 and 2019 and 1:1 age- and sex-matched controls without CP (non-CP cohort).

**Results:**

During the median follow-up period of 5.1 (interquartile range, 2.8–8.0) years, 0.56% (n = 405/72,658) of the CP cohort and 0.29% (n = 212/72,658) of the matched non-CP cohort developed lung cancer, with incidence rates of 8.3 and 4.5 per 10,000 person-years. The risk of incident lung cancer was significantly higher in the CP cohort than in the matched non-CP cohort (adjusted hazard ratio = 2.27, 95% confidence interval = 1.94–2.65). The risk of incident lung cancer was 2.45-fold and 2.10-fold higher in mild and moderate-to-severe CP cohorts than in the matched non-CP control. The risk of incident lung cancer was especially higher in the 40–59 age group, females, and never-smokers than their counterparts.

**Conclusion:**

We demonstrate that the risk of incident lung cancer is higher in individuals with CP than in those without. The risk of lung cancer was especially high in individuals with more severe CP, females, never-smokers, and obese populations.

## Introduction

Periodontal disease stems from the infection and inflammation affecting the supportive and anchoring tissues for teeth. This disease encompasses a range from mild gingivitis to more harmful periodontitis, marked by severe degradation of attachment structures like the alveolar bone and periodontal ligament, often resulting in tooth loss (1). Approximately 10% of the global population suffers from severe periodontitis (2).

Research indicates that periodontal disease, which is highly prevalent and affects approximately 90% of the world’s population (3), is correlated with an increased risk of lung cancer (4–6), even when accounting for smoking (5, 7, 8), which contributes to both periodontal disease and lung cancer. Interestingly, when evaluating this issue, in most previous studies, various types of periodontal disease, including acute periodontitis were included (1, 4–10). However, considering longstanding inflammatory conditions are more likely to be linked to carcinogenesis (11), it might be more plausible to focus on the impact of “chronic” periodontal diseases on lung cancer risk rather than evaluating any types of periodontal diseases as a whole. In addition, since most studies were conducted in Western societies (USA, Sweden, or Greece), little information is available in Asian societies (1, 10).

Accordingly, our objective was to contribute further evidence regarding the association between CP and lung cancer incidence within an Asian population by conducting an extensive analysis of a nationwide large dataset. To examine the impact of chronic inflammation and infection resulting from periodontal diseases on the incidence of lung cancer, we specifically focused on chronic periodontitis (CP) among various periodontal conditions. Additionally, we explored how the severity of CP influences the occurrence of lung cancer.

## Methods

### Data source

This study used data from the Korean National Health Insurance Service National Sample Cohort (NHIS-NSC), including a 2.2% representative sample of Korean citizens. In Korea, NHIS, a universal insurance provider managed by the government covers 97% of the Koran population, approximately 50 million citizens (12). The NHIS-NSC dataset includes information on demographic and socioeconomic variables (e.g., age, sex, income status, residential area), healthcare utilization, health screening examination findings, disease diagnosis under International Classification of Diseases-10th Revision (ICD-10) codes, drug prescription, and death. The NHIS-NSC has been widely used in various epidemiologic studies (13).

This study was approved by the Institutional Review Board of Hanyang University Hospital (IRB No. HYUH 2022-09-031). The requirement of informed consent from the participants was waived because the NHIS database was constructed after anonymization.

### Study population

A total of 1,137,861 patients were identified between January 1, 2002, and December 31, 2019. We excluded 147,520 patients who received a diagnosis of any periodontitis between January 1, 2002, and December 31, 2004. Among the remaining 990,341 patients, 565,835 patients had at least one new CP diagnosis code and 424,506 patients did not have CP diagnosis code. The index date was defined as the date when the patients received the first CP diagnosis code.

To establish the CP cohort, 147,049 patients having CP diagnosis codes twice per year and at least one treatment code within 1 year of the first diagnosis were further selected. Among those, patients who were younger than 20 years (n = 5,185) and those who were diagnosed with any type of cancer before the index date (n = 6,520) were excluded. In addition, patients who were diagnosed with lung cancer within 1 year after the index date (n = 72), those who died within 1 year after the index date (n = 208), and those who had any missing health screening data (n = 50,052) were also excluded from the CP cohort. A total of 85,012 patients were enrolled in the CP cohort.

To establish the non-CP cohort, of the initial pool of 424,506 patients without CP diagnosis code, we excluded the patients who met the same exclusion criteria applied to the CP cohort (i.e., under 20 years of age, any cancer before the index date, death or lung cancer diagnosis within 1 year after the index date, and health screening data unavailable). A total of 138,445 patients were enrolled in the non-CP cohort.

**Figure 1:**
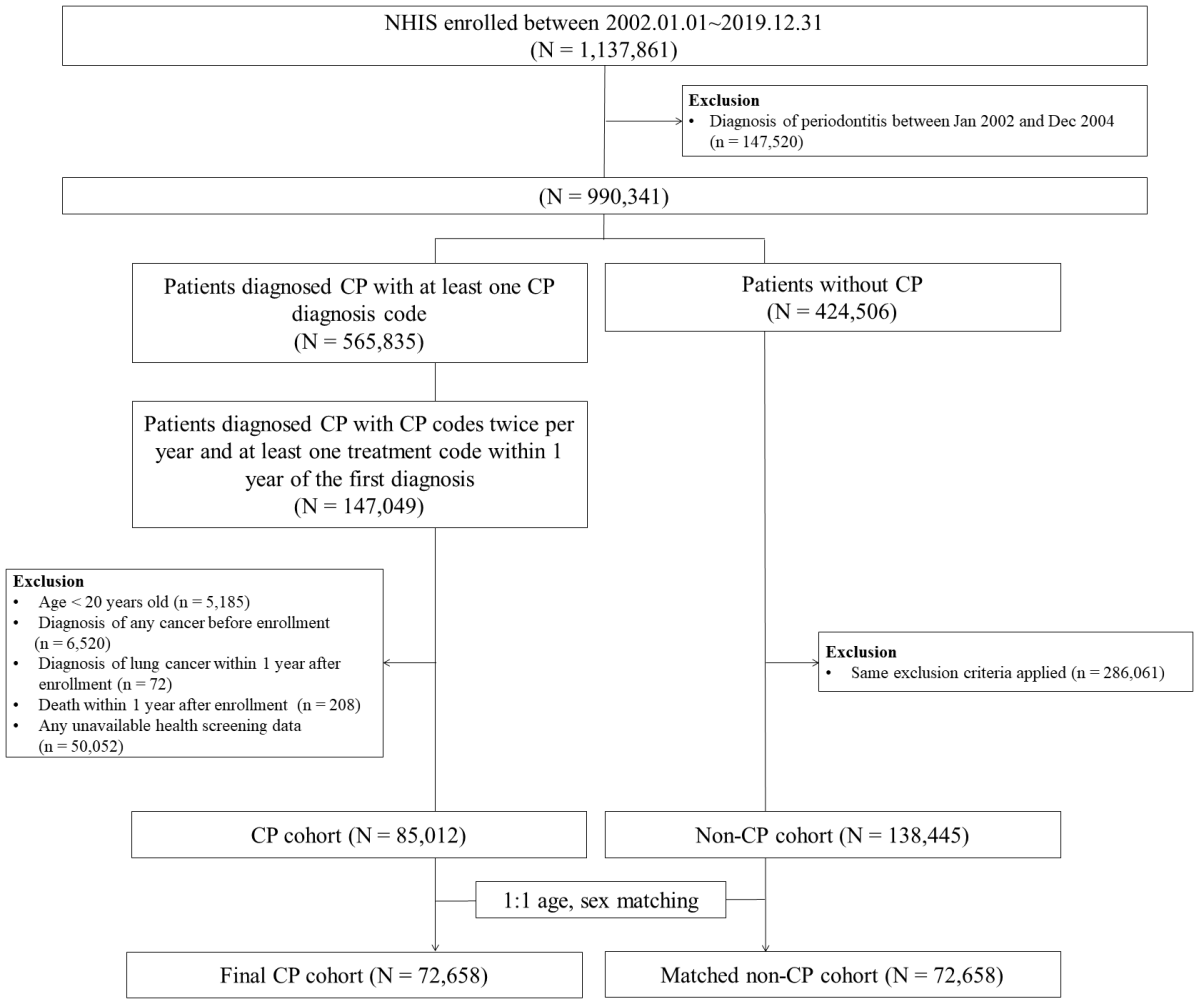
Flow chart of study population.

Thereafter, we performed 1:1 matching between the CP and non-CP cohorts based on age and sex and finally enrolled 72,658 patients for the CP and non-CP cohorts (**Figure 1**). Individuals were followed up for 1 year after study enrolment to the date of lung cancer diagnosis, death, or Dec 2019.

## Exposure

The exposure of this study was periodontitis, of which the definition required 1) diagnosis codes with periodontitis ICD-10 code (K05.3) twice per year and 2) at least one treatment code (U2232, U2233, U2240, U1010, U4411, U4412, U1051, U1052, U1701, U1702, U1081, U1082, and U1083) within 1 year of the first diagnosis.

The CP cohort was divided into 3 groups according to the severity of periodontitis (mild, moderate, and severe CP), and the severity of periodontal disease was classified according to CP-related treatment (14). Patients with periodontitis who underwent scaling (U2232, U2233) and root planning (U2240) were classified into the mild CP group, while patients who only received subgingival curettage (U1010) were classified into the moderate periodontitis group. Patients who underwent tooth extraction and severe dental treatment such as tooth extraction (U4411, U4412), periodontal flap operation (U1051, U1052), bone graft for alveolar bone defects (U1071, U1702), and guided tissue regeneration (U1081, U1082, U1083), were assigned to the severe CP group.

### Study outcomes

The primary outcome of this study was newly diagnosed lung cancer during the follow-up period. Lung cancer was defined by an ICD-10 code of C34. The study population was followed from 1 year after the index date to the date of lung cancer event, date of death, or until the end of the study period (December 31, 2019), whichever came first.

### Covariates

Household income was categorized into quartiles based on insurance premium levels (in Korea, insurance premiums are determined by income level), with those covered by Medical Aid (poorest 3%) being merged into the lowest income quartile (15–17). The lowest income quartile group was defined as “low income”. Detailed information on the patient’s smoking status was obtained through self-reported questionnaires. Smoking status was categorized into never, former, and current smokers. Body mass index (BMI) was calculated as the participant’s body weight (kg) divided by the square of the participant’s height (m^2^) and was categorized as low (<18.5), normal (18.5–22.9), overweight (23–24.9), and obese (≥25) according to the Asia-Pacific BMI criteria established by the World Health Organization. The Charlson Comorbidity Index (CCI) was categorized as 0, 1 to 2, or ≥ 3 to assess the overall comorbidity load (18).

## Statistical analysis

The baseline characteristics are presented as mean ± standard deviation (SD) for continuous variables and numbers with percentages for categorical variables. We compared the two groups using the χ2 test for categorical variables, and t-tests for continuous variables. The incidence rates of lung cancer were estimated as the number of events per 10,000 person-years. The cumulative incidence of lung cancer was presented using the Kaplan-Meier curve.

Cox proportional hazards regression analyses were used to evaluate the risk of incident lung cancer in the CP cohort versus the matched non-CP cohort. Model 1 was unadjusted, and Model 2 was adjusted for BMI and smoking status. Model 3 was further adjusted for low income, and CCI in addition to the variables adjusted in Model 2. Stratified analyses were performed based on age, sex, smoking status, BMI, and CCI. A two-sided *p-*value < 0.05 was considered statistically significant, and all analyses were conducted using SAS 9.4 (SAS Institute Inc., Cary, NC, USA) and graphs were generated using R software version 4.2.1 (Vienna, Austria).

## Results

### Baseline characteristics

The baseline characteristics of the study population are summarized in **Table 1**. The mean age of the study population was 46.5 years (SD, 13.4 years) and 50.8% were males.

**Table 1:** Baseline characteristics of study participants.

Variables	Total (n = 145,316)	CP status		
		CP cohort (n = 72,658)	Matched non-CP cohort (n = 72,658)	*P* value
**Age, mean ± SD, years**	46.5 ± 13.4	46.8 ± 13.1	46.3 ± 13.8	> 0.999
Age, years				> 0.999
20-29	13,794 (9.5)	6,897 (9.5)	6,897 (9.5)	
30-39	29,306 (20.2)	14,653 (20.2)	14,653 (20.2)	
40-49	47,690 (32.8)	23,845 (32.8)	23,845 (32.8)	
50-59	29,628 (20.4)	14,814 (20.4)	14,814 (20.4)	
60-69	15,706 (10.8)	7,853 (10.8)	78,53 (10.8)	
≥70	9,192 (6.3)	4,596 (6.3)	4,596 (6.3)	
**Sex, male**	73,844 (50.8)	36,922 (50.8)	36,922 (50.8)	> 0.999
**Low income**	35,582 (24.5)	18,104 (24.9)	17,478 (24.1)	< 0.001
Smoking				< 0.001
Never smoker	90,947 (62.6)	45,211 (62.2)	45,736 (63)	
Ex-smoker	17,957 (12.4)	9,836 (13.6)	8,121 (11.2)	
Current smoker	36,412 (25.1)	17,611 (24.2)	18,801 (25.9)	
BMI, kg/m^2^				< 0.001
< 18.5	5,812 (4.0)	2,565 (3.5)	3,247 (4.5)	
18.5–23.0	56,410 (38.8)	27,547 (37.9)	28,863 (39.7)	
23.0–25.0	33,808 (23.3)	17,563 (24.2)	16,245 (22.4)	
≥25.0	49,286 (33.9)	24,983 (34.4)	24,303 (33.5)	
Comorbidities				
Airway diseases (COPD and/or asthma)	13,829 (9.5)	7,176 (9.9)	6,653 (9.2)	< 0.001
Diabetes mellitus	12,303 (8.5)	6,557 (9)	5,746 (7.9)	< 0.001
Hypertension	25,128 (17.3)	13,136 (18.1)	11,992 (16.5)	<0.001
Dyslipidemia	21,372 (14.5)	11,958 (16.5)	9,414 (13.0)	<0.001
CKD	381 (0.3)	180 (0.3)	201 (0.3)	0.281
CCI score				< 0.001
0	88,787 (61.1)	42,896 (59.1)	45,891 (63.2)	
1	33,530 (23.1)	17,390 (23.9)	16,140 (22.2)	
2	12,811 (8.8)	6,922 (9.5)	5,889 (8.1)	
≥ 3	10,188 (7.0)	5450 (7.5)	4,738 (6.5)	
CP severity				–
Mild	N/A	38,292 (52.7)	N/A	
Moderate	N/A	26,073 (35.9)	N/A	
Severe	N/A	8,293 (11.4)	N/A	

Data are presented as mean ± SD or number (percentage).

CP, chronic periodontitis; BMI, body mass index; COPD, chronic obstructive pulmonary disease; CKD, chronic kidney disease; CCI, Charlson comorbidity index, N/A, not applicable.

In terms of age and sex, both groups were well-balanced (*p* > 0.999). The proportion of ex-smokers, obesity, and comorbidities except for chronic kidney disease (*p* = 0.281), was significantly higher in the CP cohort than in the non-CP cohort (*p* < 0.001 for all). Additionally, the CCI score was higher in the CP cohort than in the non-CP cohort (*p* < 0.001, *p* for trend < 0.001).

In the CP cohort, the proportion of mild, moderate, and severe CP was 52.7% (n = 38,292), 35.9% (n = 26,073), and 11.4% (n = 8,293), respectively.

### Incidence and risk of lung cancer

**Figure 2:**
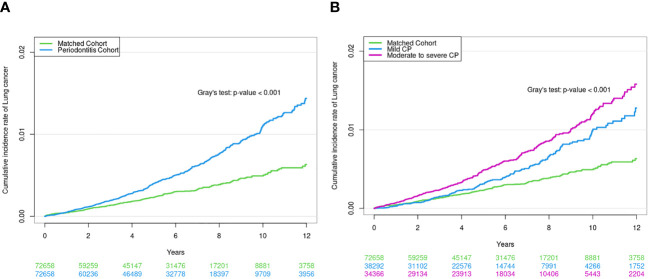
**(A)** Cumulative incidence of lung cancer in CP cohort and matched non-CP cohort. **(B)** Cumulative incidence of lung cancer according to CP severity.

During the median follow-up period of 5.1 (interquartile range, 2.8–8.0) years, 0.56% (405/72,658) of the CP cohort and 0.29% (212/72,658) of the matched non-CP cohort developed lung cancer, with incidence rates of 8.3 and 4.5 per 10,000 person-years, respectively. Even after adjusting for potential confounders, the risk of incident lung cancer was also significantly higher in the CP cohort than in the matched non-CP cohort: unadjusted hazard ratio (HR) = 2.06, 95% confidence interval [CI] = 1.80–2.37; adjusted HR in Model 2 = 2.29, 95% CI = 1.96–2.67; adjusted HR in Model 3 = 2.27, 95% CI 1.94–2.65) (**Table 2**). Similarly, a cumulative incidence plot depicts a significantly higher incidence of lung cancer in the CP cohort than in the matched non-CP cohort (a log-rank *p* < 0.001; **Figure 2A**).

**Table 2:** Subdistribution incidence and HRs for Incident lung cancer in the CP and matched non-CP cohort.

	Participants No.	Lung cancer	Duration (PY)	Incident rate (/10,000 PY)	Model 1 HR (95% CI)	Model 2 HR (95% CI)	Model 3 HR (95% CI)
By CP status							
Matched non-CP cohort	72,658	212	475,608.3	4.5	Reference	Reference	Reference
CP cohort	72,658	405	487,722.0	8.3	2.06 (1.80, 2.37)	2.29 (1.96, 2.67)	2.27 (1.94, 2.65)
By CP status and severity							
Matched non-CP cohort	72,658	212	475,608.3	4.5	Reference	Reference	Reference
Mild CP cohort	38,292	162	240,828.0	6.7	1.86 (1.51, 2.28)	2.07 (1.64, 2.61)	2.10 (1.66, 2.65)
Moderate-to-severe CP cohort	34,366	243	246,894.0	9.8	2.23 (1.86, 2.68)	2.49 (2.01, 3.07)	2.45 (1.97, 3.04)

Model 1 was unadjusted.

Model 2 was adjusted for BMI and smoking status.

Model 3 was adjusted for BMI, smoking status, low income, and CCI.

HR, hazard ratio; CP, chronic periodontitis; PY, person-years; CI, confidence interval; BMI, body mass index; CCI, Charlson Comorbidity index.

When CP was classified according to severity (control *vs*. mild *vs*. moderate-to-severe), 0.42% (162/38,292) of the mild CP cohort and 0.71% (243/34,366) of the moderate-to-severe CP cohort developed lung cancer, with incidence rates of 6.7 and 9.8 per 10,000 person-years, respectively. The risk of incident lung cancer was 2.10-fold (95% CI, 1.66–2.65) and 2.45-fold (95% CI, 1.97–3.04) higher in mild and moderate-to-severe CP cohorts than in matched non-CP controls (fully adjusted Model 3), respectively (**Table 2**). The cumulative incidence plot depicts similar results (a log-rank *p* < 0.001; **Figure 2B**).

### Subgroup analysis

Stratified analyses regarding the risk of lung cancer among patients with CP compared with subjects without CP are shown in **Table 3**. Age, sex, and smoking had a significant interaction on the association of CP with lung cancer development (*p* for interaction < 0.001 for all). The risk of incident lung cancer was higher in the 40–59 age group, females, and never-smokers compared to their counterparts.

**Table 3:** Subgroup analysis for the incidence of lung cancer the CP and matched non-CP cohort.

Subgroups	CP	Model 1 HR (95% CI)	Model 2 HR (95% CI)	Model 3 HR (95% CI)
Age, years				
20 – 39	No	Reference	Reference	Reference
	Yes	4.33 (1.52, 12.34)	5.10 (1.40, 18.63)	3.10 (0.88, 10.94)
40 – 59	No	Reference	Reference	Reference
	Yes	5.00 (3.52, 7.10)	5.27 (3.61, 7.71)	5.27 (3.62, 7.67)
≥ 60	No	Reference	Reference	Reference
	Yes	1.46 (1.25, 1.70)	1.62 (1.36, 1.92)	1.61 (1.35, 1.91)
*p* for interaction		< 0.001	< 0.001	< 0.001
Sex				
Male	No	Reference	Reference	Reference
	Yes	1.59 (1.37, 1.86)	1.80 (1.51, 2.15)	1.81 (1.51, 2.16)
Female	No	Reference	Reference	Reference
	Yes	4.13 (3.00, 5.68)	4.35 (3.07, 6.16)	4.29 (3.01, 6.10)
*p* for interaction		< 0.001	< 0.001	< 0.001
Smoking				
Never-smoker	No	Reference	Reference	Reference
	Yes	3.52 (2.67, 4.65)	3.56 (2.68, 4.72)	3.52 (2.65, 4.69)
Ever-smoker	No	Reference	Reference	Reference
	Yes	1.46 (1.15, 1.87)	1.54 (1.19, 1.98)	1.58 (1.21, 2.07)
*p* for interaction		< 0.001	< 0.001	< 0.001
BMI				
< 22.9	No	Reference	Reference	Reference
	Yes	1.76 (1.31, 2.36)	1.94 (1.39, 2.69)	1.99 (1.43, 2.78)
23.0 – 24.9	No	Reference	Reference	Reference
	Yes	2.00 (1.14, 3.52)	2.96 (1.38, 6.33)	2.61 (1.15, 5.93)
≥ 25.0	No	Reference	Reference	Reference
	Yes	3.00 (1.89, 4.76)	3.39 (1.99, 5.77)	5.13 (2.74, 9.62)
*p* for interaction		0.221	0.187	0.190

Model 1 was unadjusted.

Model 2 was adjusted for BMI and smoking status.

Model 3 was adjusted for BMI, smoking status, low income, and CCI.

CP, chronic periodontitis; HR, hazard ratio; CI, confidence interval; BMI, body mass index; CCI, Charlson Comorbidity index.

## Discussion

This study provides longitudinal evidence from an Asian population, using nationwide data with a median follow-up of 5 years to explore the association between CP and lung cancer risk. We investigated whether CP is a predisposing factor for the development of lung cancer, and which factors are related to an increased risk of lung cancer among individuals with CP. The results showed that the CP cohort had a lung cancer incidence rate of 8.3 per 10,000 person-years, which is 2.3-fold higher compared with the matched non-CP cohort. There was a significant dose-dependent relationship between CP severity and lung cancer risk. The risk of incident lung cancer was 2.10-fold and 2.45-fold higher in mild and moderate-to-severe CP cohorts than in matched non-CP controls. Specifically, certain groups including those aged 40–59, females, and never smokers exhibited a higher susceptibility to lung cancer development.

Previous meta-analyses showed that the risk of lung cancer is 1.24 to 1.40-fold higher in individuals with periodontal disease compared to those without periodontal disease (6, 8, 19). However, in these meta-analyses, various types of periodontal disease were included. Considering longstanding inflammatory conditions are more likely to be linked to carcinogenesis, it might be more plausible to hypothesize chronic periodontal disease may be associated with increased lung cancer risk. As we assumed, the strength of the association between CP and lung cancer risk in our study (aHR = 2.3) was higher than that of previous meta-analyses as well as a previous result that was performed in a similar study population in Korea (1). Besides this issue, we also like to emphasize that there is not enough evidence on the association between periodontal disease and the risk of lung cancer in the Asian population. Since most studies included in the previous meta-analyses were conducted in Western society (4, 5, 8, 9), little information has been available on the Asian population (1, 10), needing supporting data on this population. From this view, our comprehensive analyses that considered numerous confounders (e.g., demographics, smoking status, and comorbid conditions) would be valuable by providing supporting information on this issue in Asian population.

The relationship between CP and lung cancer can be explained by the following hypotheses. First, the oral microbiome may influence lung cancer incidence. With mounting evidence highlighting the connection between human microorganisms and malignant tumors, it has become evident that a unique microbiome exists in the lungs, potentially exerting influence on the development of lung cancers (20). Recent findings from data using three prospective cohort studies in the US reinforce this notion (21). It has been proposed that these microorganisms predominantly originate from the oral microbiome through micro-aspiration of oral fluids (22). The potential mechanisms behind this phenomenon involve an increase in the inflammatory environment, which could promote carcinogenesis, microbial influences on host metabolism, and genotoxicity. Yan et al. discovered significantly elevated counts of *Veillonella* and *Capnocytophaga* species in the saliva of lung cancer patients (23). One study in ARIC study included 4,263 cancer-free participants with previously measured antibodies of oral bacteria, and 1,287 participants from whom subgingival plaque was collected (9). They reported positive associations with lung cancer for *Fusobacterium nucleatum*, *Aggregatibacter actinomycetemcomitans*, and *Porphyromonas gingivalis* counts, and significant positive associations were found for the count to antibody ratio for *Prevotella intermedia* and *P. gingivalis* (9).

*P. gingivalis*-stained sections exhibited significantly higher frequency and intensity in cancerous tissues of small cell lung cancer, lung adenocarcinoma, and lung squamous cell carcinoma, when compared to adjacent lung tissues (24). Furthermore, by evaluating immunoglobulin G (IgG) antibodies specific to *P. gingivalis* in the serum of lung cancer patients, researchers have identified a positive correlation between the antibody levels and the risk of developing lung cancer, as compared to a cohort of healthy controls (25). Similar to the findings related to *P. gingivalis*, a positive correlation has been identified between serum levels of IgG antibodies specific to *F. nucleatum* and the incidence of lung cancer (9). This alignment between antibody levels and lung cancer highlights a potential association between *F. nucleatum* and the pathogenesis of lung cancer.

Second, although the exact carcinogenic mechanisms remain unclear, periodontal disease, along with the release of inflammatory factors into the blood, might impact the incidence of lung cancer. One study investigated the relationship between immune surveillance mechanisms and periodontitis in cancer patients (26). In this study, the peripheral blood concentration of IL-6 was significantly higher in cancer patients than in non-cancer patients, and it was even higher in cancer patients with periodontitis compared to those without. Additionally, the peripheral blood proportion of regulatory T cells was significantly higher in cancer patients with periodontitis than in other groups (including cancer patients without periodontitis, non-cancer patients with periodontitis, and non-cancer patients without periodontitis). These results suggest that the presence of periodontitis might synergistically contribute to cancer development and progression. Other studies have also described a positive relationship between CP and systemic inflammation (27, 28), as well as the association between systemic inflammation and lung cancer development. CP leads to increased level of IL-6 as well as C-reactive protein, IFN-γ, and IL-1β (27–29). These periodontal pathogens and inflammation products enter the bloodstream, triggering a systemic inflammatory response (30). Previous studies have linked elevated levels of C-reactive protein, IL-6, IFN-γ, and IL-1β to an increased risk of lung cancer (31, 32).

In our study, a higher risk of lung cancer in the CP group versus controls was related to females and never-smokers. In females populations, it is known that gastroesophageal reflux disease (GERD) is more common than their counterparts (33); Considering CP-associated changes in oral microbiota might influence lung microbiota, which can influence the occurrence of lung cancer, through micro-aspiration of oral microbiota into the lungs. Interestingly, the risk of lung cancer in subjects with CP versus those without CP was more substantial among never-smokers. Although the reasons are not fully explainable, we carefully suggest that the impact of smoking on the development of lung cancer was more significant than that of CP, and, as a result, the influence of CP might have been relatively obscured among smokers.

We would like to suggest one important clinical implication of our study. Current lung cancer screening strategies do not reflect risk factors other than smoking status and age (34). However, numerous studies have revealed there are various important risk factors for lung cancers, such as air pollution, infectious/inflammatory conditions (e.g., tuberculosis, bronchiectasis, connective tissue diseases, chronic periodontitis, etc.) (35–37), which might be more important for lung cancer not related to smoking. From this view, our study results would be very helpful for building future lung cancer prediction models or strategies, although more studies are performed on which variables need to be included to build a cost-effective prediction model for lung cancer.

Our study has two important limitations. First, since this study was conducted in a Korean population, it may be difficult to generalize the study results to other ethnic and country groups. Second, CP, lung cancer, and comorbidities were determined using ICD-10 codes. Thus, there might be over or underestimation of the diagnoses.

In conclusion, a nationwide longitudinal database demonstrates that the risk of incident lung cancer is higher in individuals with CP than in those without, and the more severe the CP, the higher the risk of lung cancer. Additionally, our results suggest that CP may further influence lung cancer development in females and never-smokers. Providing care and treatment for CP in these populations will be particularly important.

## Data availability statement

The raw data supporting the conclusions of this article will be made available by the authors, without undue reservation.

## Ethics statement

This study was approved by the Institutional Review Board of Hanyang University Hospital (IRB No. HYUH 2022-09-031). The studies were conducted in accordance with the local legislation and institutional requirements. The ethics committee/institutional review board waived the requirement of written informed consent for participation from the participants or the participants’ legal guardians/next of kin the NHIS database was constructed after anonymization.

## Author contributions

B-GK: Conceptualization, Data curation, Formal analysis, Methodology, Validation, Writing – original draft, Writing – review & editing. HL: Conceptualization, Data curation, Formal analysis, Methodology, Writing – original draft, Writing – review & editing, Validation. S-KL: Data curation, Formal analysis, Methodology, Visualization, Writing – review & editing, Investigation, Writing – original draft. SP: Data curation, Writing – review & editing, Investigation, Validation, Writing – original draft. S-HY: Data curation, Writing – review & editing, Investigation, Writing – original draft. C-JP: Data curation, Writing – review & editing, Investigation, Writing – original draft. YY: Data curation, Writing – review & editing, Investigation, Writing – original draft. TP: Data curation, Writing – review & editing, Investigation, Writing – original draft. J-YM: Data curation, Writing – review & editing, Investigation, Writing – original draft. T-HK: Data curation, Writing – review & editing, Investigation, Writing – original draft. JS: Data curation, Writing – review & editing, Investigation, Writing – original draft. S-HK: Data curation, Investigation, Writing – review & editing, Writing – original draft. HY: Writing – original draft, Writing – review & editing, Data curation, Investigation, Methodology, Supervision. DP: Data curation, Formal analysis, Investigation, Methodology, Supervision, Writing – original draft, Writing – review & editing.
